# IdeS: A Bacterial Proteolytic Enzyme with Therapeutic Potential

**DOI:** 10.1371/journal.pone.0001692

**Published:** 2008-02-27

**Authors:** Björn P. Johansson, Oonagh Shannon, Lars Björck

**Affiliations:** Division of Infection Medicine, Department of Clinical Sciences, Biomedical Center (BMC), Lund University, Lund, Sweden; Rikshospitalet University of Oslo, Norway

## Abstract

**Background:**

IdeS, a proteinase from *Streptococcus pyogenes*, cleaves immunoglobulin (Ig)G antibodies with a unique degree of specificity. Pathogenic IgG antibodies constitute an important clinical problem contributing to the pathogenesis of a number of autoimmune conditions and acute transplant rejection. To be able to effectively remove such antibodies is therefore an important clinical challenge.

**Methodology/Principal Findings:**

IdeS was found to specifically and efficiently cleave IgG in human blood *in vitro* (20 µg of IdeS caused a complete degradation of IgG in one ml of human whole blood in 15 minutes) and to clear IgG from the blood stream of rabbits *in vivo* (no IgG was detected six hours following an intravenous injection of 5 mg of IdeS) without any side effects. In a mouse model of immune thrombocytopenic purpura (ITP), polyclonal IgG antibodies against platelet surface antigens were used to induce a lethal disease. These profoundly thrombocytopenic animals were treated and cured by a single injection of IdeS.

**Conclusions/Significance:**

Novel information is provided concerning the IgG-cleaving activity of IdeS *in vitro* and *in vivo*. The highly specific and rapid elimination of IgG *in vivo*, the dramatic effect in a mouse model of ITP, and the lack of side effects in the treated animals, indicate that IdeS could also be used to treat IgG-driven diseases in humans.

## Introduction

Antibodies play an important role in the defence against invading microorganisms. Among the five classes of antibodies, immunoglobulin (Ig)G is the quantitatively dominating class, and apart from albumin the most abundant protein in human blood (7–16 mg/ml blood plasma). Besides its protective role, IgG is also involved in disease. It is estimated that autoimmune diseases affect about five percent of people [1], and in many of these conditions (rheumatoid arthritis, systemic lupus, myasthenia gravis, immune (or idiopathic) thrombocytopenic purpura (ITP), Goodpasture's syndrome, etc) IgG autoantibodies reacting with human molecules contribute to the pathogenesis. They also cause acute transplant rejection, bind and inactivate life-saving hemophilia factors, whereas overproduction of IgG in patients with multiple myeloma is associated with a hyperviscosity syndrome. In these conditions removal of pathogenic IgG antibodies from the blood circulation represents a logical therapeutic strategy, and extracorporeal immunoadsorption where patient plasma is passed over columns that bind IgG, has indeed proven to be beneficial in several autoimmune conditions [2]–[7]

*Streptococcus pyogenes*, a significant bacterial pathogen, secretes two enzymes showing remarkable specificity for IgG; EndoS and IdeS. EndoS (Endoglycosidase in *Streptococcus pyogenes*) efficiently and specifically hydrolyzes the functionally important N-linked glycan of IgG [8], and treatment with EndoS abrogates the pathogenic activity of IgG in mouse models of autoimmune disease [9], [10]. IdeS (Immunoglobulin G-degrading enzyme of *Streptococcus pyogenes*) is a cysteine proteinase which cleaves IgG with a unique degree of specificity in the hinge region [11], [12], and in a recent study the enzyme was found to block the development of IgG-induced arthritis in a mouse model of disease [13]. In the present work we further investigate the potential of IdeS for the treatment of conditions involving pathogenic IgG antibodies. The results demonstrate that the enzyme *in vitro* efficiently cleaves IgG in human whole blood, removes IgG from the blood circulation of rabbits without any side effects, and cures mice from lethal IgG-induced thrombocytopenia.

## Results and Discussion

**Figure 1:**
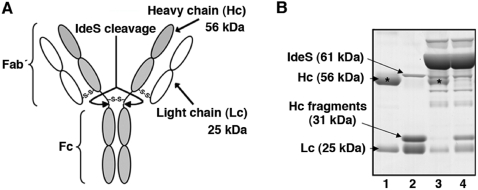
IdeS cleaves IgG in human blood. (A) Structure of IgG. The IdeS cleavage sites are indicated. (B) The following samples were separated by SDS-PAGE. Lane 1: Five µg of human polyclonal IgG in 10 µl PBS. Lane 2: Five µg of human polyclonal IgG and 1 µg of IdeS in 10 µl PBS (IgG and IdeS were preincubated for three hours at 37°C before SDS-PAGE). Lane 3: Ten µl of plasma from human blood diluted 1∶50 in PBS Lane 4: One hundred µl of human blood was preincubated with 1 µg of IdeS for three hours at 37°C. The plasma from this sample (containing approximately 20 µg/ml) was diluted 1∶50 in PBS, and 10 µl of this material was separated in lane 4. The asterisk indicates the IgG heavy chain.

IdeS [11], [12], also called Mac-1 [14], is a proteolytic enzyme produced by the bacterial pathogen *Streptococcus pyogenes*, which cleaves IgG with a unique degree of specificity [11], [15]. Before IdeS can cleave IgG in the heavy chain (see Fig. 1A) the enzyme has to bind to the Fc region of the antibody, and the remarkable specificity of IdeS lies in this protein-protein interaction [15]. The IgG molecule is composed of two heavy and two light chains which are held together by disulfide bonds (Fig. 1A). IdeS (throughout this study a 61 kDa IdeS fusion protein with GST was used) cleaves IgG in the heavy chains (Fig. 1A), which generates one F(ab′)_2_ and two monomeric Fc fragments. When these fragments are separated by SDS-PAGE under reducing conditions (to break the disulfide bonds), the heavy chains give rise to bands of 25 (the Fc monomers) and 31 kDa (the remaining part of the heavy chains), and the light chains dissociate from the (Fab′)_2_ fragments. Since the molecular mass of the light chains is also 25 kDa, SDS-PAGE of IgG completely cleaved by IdeS will generate two bands of 25 and 31 kDa, respectively, whereas SDS-PAGE of uncleaved IgG under reducing conditions produces two bands corresponding to intact heavy (56 kDa) and light (25 kDa) chains (Fig. 1B, lanes 1 and 2).

**Figure 2:**
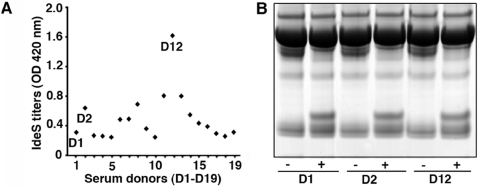
IgG antibodies against IdeS in human sera do not interfere with the enzymatic activity. (A) Antibody titers against IdeS in serum samples from nineteen healthy donors (D1–D19). (B) SDS-PAGE analysis of serum samples untreated (−) or preincubated with IdeS (20 µg/ml serum) for three hours at 37°C (+).

To investigate the IgG-cleaving activity of IdeS in blood, whole human blood was incubated with IdeS and a complete degradation of IgG was obtained (Fig. 1B, lanes 3 and 4). Most individuals have been infected with *S. pyogenes* bacteria and antibodies against streptococcal proteins, including IdeS, are very common in the human population [16]. It has been reported that convalescent-phase sera from patients with *S. pyogenes* infections in some cases partially inhibit the activity of IdeS [17]. In that study, the concentration of IdeS was approximately a thousand fold lower as compared to the concentration used here. It should also be mentioned that streptokinase, a plasminogen activator and like IdeS a protein of *S. pyogenes*, has been used therapeutically as a thrombolytic agent for decades. In these patients antibodies against streptokinase are often present, but seldom cause problems unless the patients are treated repeatedly. In addition, it could be that IdeS by cleaving IgG antibodies, including IgG directed against itself, is less sensitive to neutralizing IgG antibodies in comparison to streptokinase and other protein antigens. However, neutralizing antibodies against IdeS are of course a potential problem, and we therefore tested the IgG titers against IdeS in nineteen healthy individuals. As expected, all had anti-IdeS IgG antibodies (Fig. 2A), but the cleavage of IgG by IdeS was not affected even in the sera with the highest titers against IdeS (Fig. 2B). The results show that these antibodies do not inactivate the IgG-cleaving activity of IdeS when used at a concentration (20 µg IdeS/ml plasma) that completely cleaves IgG in human blood (Fig. 1B, lane 4). This does of course not exclude the possibility that repeated injections of IdeS in humans, as is the case with streptokinase, may cause problems in some patients due to neutralizing antibodies or allergic reactions.

**Figure 3:**
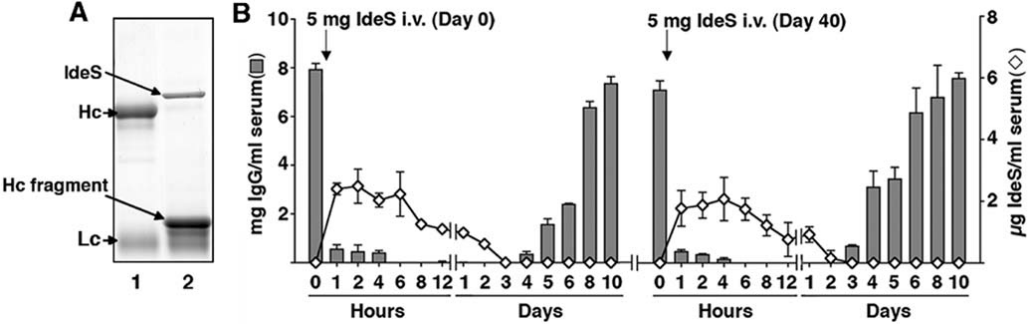
*Invivo* cleavage and removal of IgG from the blood circulation of rabbits injected with IdeS. (A) SDS-PAGE of rabbit polyclonal IgG (5 µg in 10 µl PBS) alone (lane 1), or preincubated with IdeS (5 µg IgG and 1 µg IdeS in 10 µl PBS) for three hours at 37°C (lane 2). Bands corresponding to IdeS (61 kDa), IgG heavy chains (Hc, 56 kDa), IdeS-generated Hc fragments (31 kDa) and IgG light chains ( Lc, 25 kDa), are indicated. (B) Levels of IgG (grey bars) and IdeS (⋄) in serum samples from a rabbit injected i.v. with IdeS (5 mg diluted in 2.5 ml PBS). IgG was determined by ELISA and IdeS by Western blotting and chemoluminescence in a Chemidoc XRS Imaging system. Samples were analyzed three times and mean values±SD are indicated.

To test IdeS toxicity and IgG cleavage *in vivo* under conditions resembling a clinical situation, we needed to find an animal species in which all IgG subclasses, as in humans [11], are cleaved by IdeS. Only partial cleavage was obtained with polyclonal mouse and rat IgG (data not shown), whereas polyclonal rabbit IgG was found to be completely degraded (Fig. 3A). The total degradation of both human and rabbit IgG, and the fact that multiple serum samples are easier to obtain from rabbits than from mice, made us choose rabbits for the initial *in vivo* experiments. Before IdeS was injected into rabbits, we titrated the IgG-cleaving activity of the enzyme in rabbit and human whole blood *in vitro*. A complete cleavage of IgG in 1 ml rabbit blood in 15 minutes at 37°C, was found to require 5 µg of IdeS, which corresponds to 1.6 µg IdeS/mg rabbit IgG. In human blood 20 µg/ml (3.9 µg IdeS/mg IgG) was required for total IgG degradation, *i.e.* IdeS cleaves rabbit IgG more effectively than human IgG.

A first rabbit was injected intravenously (i.v.) with 5 mg (corresponds to approx. 10 µg/ml blood) of IdeS on day 0 and day 40. Blood samples were drawn at different time points and analyzed for IgG and IdeS content (Fig. 3B, left panel). The effect of IdeS was rapid and efficient; within six hours intact IgG could no longer be detected in the blood stream (Fig. 3B). The clearance of the F(ab^1^)_2_ fragments generated by the IdeS treatment, was analyzed by following the presence of the 31 kDa heavy chain fragment (see Fig. 1 and Fig. 3A) in the rabbit blood samples by SDS-PAGE (data not shown). This fragment, and thereby the F(ab^1^)_2_ fragments, was cleared from the circulation within two days. After 4–5 days IgG started to reappear and was back to normal levels on day 10. IdeS was not detectable after day three (Fig. 3B, left panel), and since the GST-IdeS fusion protein used here is 61 kDa (Fig. 1B), the protein is most probably cleared via the kidneys analogous to other proteins in plasma with a molecular mass below that of albumin (69 kDa). The IdeS injection was repeated on day 40, and the same pattern was seen (Fig. 3B, right panel). The cleavage and clearance of IgG, and the clearance of IdeS, were similar in five additional rabbits, and no apparent side effects were observed in any of the IdeS-treated rabbits. Two of the rabbits, each injected i.v. on six different occasions with 5 mg of IdeS during a period of eight months, were sacrificed. Histo-pathological analysis of the organs revealed normal heart, lung, liver, spleen, and kidney tissue.

**Figure 4:**
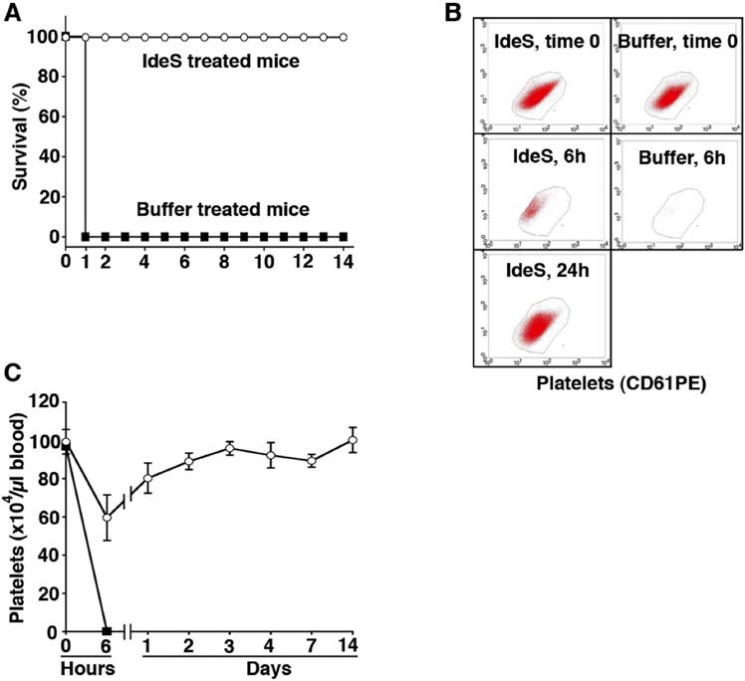
IdeS cures mice from lethal IgG-induced thrombocytopenia. (A) Fourteen BALB/C mice were injected i.p. with a lethal dose of purified polyclonal rabbit IgG antibodies (1 mg IgG/ mouse, diluted in 0.25 ml PBS) raised against mouse platelets. Thirty minutes later, seven mice were treated i.p. with 0.5 mg IdeS in 0.25 ml PBS and seven with 0.25 ml PBS alone. In another experiment, twelve Balb/c mice were injected i.p. with the lethal dose of IgG. After thirty minutes, six mice were given 0.5 mg IdeS in 0.25 ml PBS, and six were given 0.25 ml PBS alone. In this case, the injections were given i.v. The thirteen IdeS treated mice (○) all survived, whereas the thirteen PBS treated mice (▪) all died within 24 hours. (B) Blood samples from the twelve mice injected with the lethal dose of purified rabbit IgG and treated i.v. with IdeS or PBS alone (six mice in each group), were analyzed using flow cytometry of the platelet population. Samples from two representative animals, one injected with IdeS and one with PBS, are shown. (C) The number of platelets in the blood samples from the twelve animals treated i.v. was counted manually; six treated with IdeS (○) and six with PBS (▪). Error bars indicate ±SEM.

**Figure 5:**
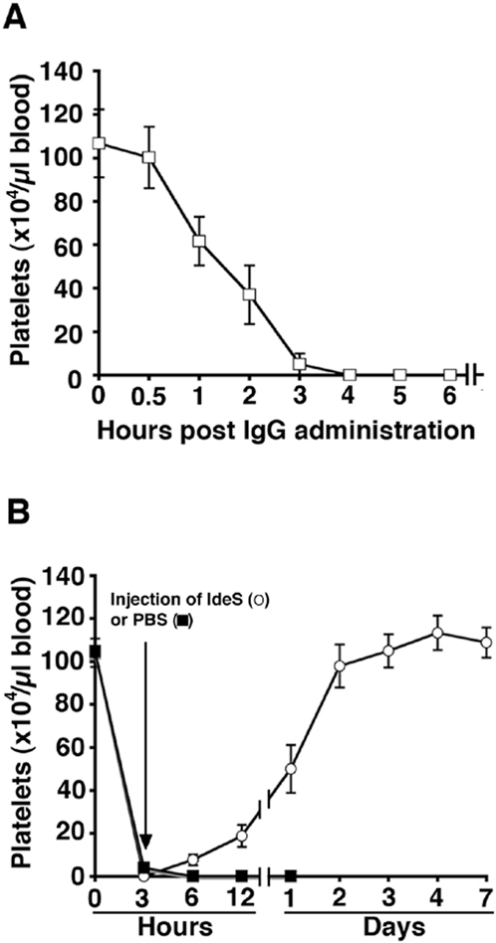
IdeS treatment of thrombocytopenia is effective also when initiated at low platelet levels. (A) Blood samples were taken at regular time intervals from three mice injected i.p. with a lethal dose of rabbit anti-mouse platelet IgG antibodies (1 mg IgG/mouse), and the number of platelets was determined by manual counting. (B) An additional ten mice were injected with the lethal dose of IgG. At three hours, five mice were treated i.v. with IdeS (0.5 mg in 0.25 ml PBS/mouse), while the other five received PBS alone (0.25 ml i.v/mouse). The PBS treated animals remained thrombocytopenic and died within 18–30 hours of IgG administration, whereas the IdeS treated animals all recovered their platelet levels and survived. These animals were sacrificed on day 14.

The very rapid and efficient cleavage and removal of IgG from the circulating blood of rabbits by IdeS, suggest that the enzyme could be used for the treatment of acute autoimmune conditions such as acute transplant rejection, Goodpasture's syndrome, severe relapses of systemic lupus, and immune thrombocytopenic purpura (ITP). In patients with ITP, polyclonal IgG autoantibodies react with different glycoproteins at the platelet surface [18]. IgG bound to the platelet surface will expose their Fc regions and bind to Fcγ receptors displayed by macrophages. As a result IgG-coated platelets are rapidly phagocytosed by macrophages (predominantly in the spleen and the liver), and the lifespan of platelets that enter the blood where such autoantibodies are present, is only a few hours compared to a normal life span of ten days. This rapid elimination exceeds the rate of production of platelets in the bone marrow, and leads to an acute or chronic thrombocytopenia and associated bleeding disorder. A mouse model which closely mimics ITP is available, where rabbits are immunized with mouse platelets and polyclonal IgG purified from the sera of these rabbits, are injected into mice [19]. When the antibodies bind to antigens on the platelet surface, the mice rapidly develop thrombocytopenia and petechiael haemorrhaging, and they eventually succumb to massive internal bleeding. To test the effect of IdeS in an animal model of an acute autoimmune condition, this mouse model was very relevant. The fact that polyclonal rabbit IgG is used to induce the disease is also a great advantage, since IdeS has the potential capacity to cleave all the administered rabbit IgG antibodies. Thus, polyclonal IgG was purified from a rabbit antiserum raised against mouse platelets, and the purified rabbit anti-mouse platelet IgG antibodies were injected intraperitoneally (i.p.) into mice. As previously reported the antibodies rapidly induced a severe thrombocytopenia in the animals [19], [20]. Following a pilot experiment where 1.0 mg of the purified rabbit IgG was found to cause a lethal thrombocytopenia in the treated mice within 24 hours, fourteen mice were each injected i.p. with this dose of IgG. Thirty minutes later seven of the mice were injected, also i.p., with 0.5 mg IdeS diluted in PBS buffer, and seven with buffer alone. An IgG control was not necessary since previous studies using this model have shown that administration of polyclonal IgG purified from non-immunized or PBS-immunized rabbits, does not affect platelet numbers or the survival of mice [19], [20]. In the PBS group all animals died from severe bleeding within 24 hours. In contrast, the IdeS treated mice showed no signs of disease and they were sacrificed after two weeks of observation. To investigate the effect of IdeS when the enzyme is administered through another route than the IgG antibodies, twelve additional mice were given the lethal IgG dose i.p. After thirty minutes, six were treated with 0.5 mg IdeS in PBS, but this time the enzyme was injected i.v.. The remaining six mice were given PBS alone, also i.v. Again the buffer treated animals were all dead within 24 hours, whereas the IdeS treated animals remained healthy and unaffected until sacrificed two weeks later. The results, summarized in Fig. 4A, show that thirteen mice treated i.p. or i.v. with IdeS, all survive a lethal dose of IgG antibodies. This effect is explained by the data shown in Fig. 4B and C. Blood samples from the mice injected i.v. with IdeS or PBS alone, were analyzed by flow cytometry. The platelets in the samples were also counted manually. In the IdeS treated mice, the number of platelets was reduced by approximately forty percent at six hours, but was back to normal levels after 2–3 days. The initial drop in platelet numbers also in IdeS treated mice, demonstrates that the IgG antibodies rapidly bind to the platelets and induce the lethal condition. However, although the disease process has already started it is counteracted when IdeS appears in the circulation. In contrast, no platelets could be detected in the buffer treated animals after six hours, and they died 3–18 hours later. In a final set of experiments, we investigated whether IdeS is effective also when the enzyme is administered later during the time course of the thrombocytopenia. Three mice where injected with the rabbit antibodies, and the number of platelets were counted in order to determine the time point when profound thrombocytopenia occurs. After three hours no platelets were present in the blood of two of the mice, and in the third mouse the number was reduced by more than 90% (Fig. 5A). Subsequently, ten additional mice were injected i.p. with the lethal dose of IgG antibodies (1 mg/mouse). At three hours, nine of the animals had no circulating platelets and one had less than 15% of its original number. At this point the mice were divided into two groups of five animals each. One group was treated i.v. with IdeS (none of the animals in this group had any circulating platelets), and the mice of the other group received PBS alone. In this latter group four animals had no circulating platelets and one had less than 15% of its original level. The effect was dramatic; PBS treated mice remained thrombocytopenic and died 15–27 hours later, whereas the IdeS treated mice all recovered their platelet levels and survived (Fig. 5B). These experiments show that IdeS is capable of rescuing the animals also when they have developed a profound and a otherwise lethal thrombocytopenia.

The results of this study demonstrate that IdeS efficiently degrades IgG in human whole blood *in vitro*, cleaves and clears IgG from the blood circulation of rabbits *in vivo*, and cures mice from lethal IgG-induced thrombocytopenia. Before IdeS can be used in patients, regulatory issues have to be addressed, but the present work indicated that the enzyme has a clinical potential. The unique and astonishing specificity of IdeS further underlined by this work, means that the other classes of antibodies are not cleaved [11], which reduces the risk of creating a serious Ig deficiency (none of the rabbits treated with IdeS showed any signs of infection). Cleaved and removed IgG antibodies could also be replaced as soon as IdeS has been cleared from the circulation. Such replacement together with an immunosuppressive regimen, may block or postpone the reappearance of pathogenic IgG antibodies. On the other hand, if the clinical condition should require a longer period of IgG deprivation, the half life of IdeS in the blood stream can be prolonged by producing a larger IdeS fusion protein. Finally, the lack of side effects and the efficient and reproducible activity when injected *in vivo*, also indicate a therapeutic potential of IdeS.

## Materials and Methods

### Recombinant expression and purification of IdeS

The expression of recombinant GST-IdeS in *Escherichia coli* has previously been described [11]. The fusion protein was purified on Glutathione Sepharose 4 Fast Flow (Amersham Biosciences) according to standard protocols, dialyzed against phosphate buffer saline (PBS), and sterile filtered through a 0.22 µm Millex-GP filter unit (Millipore Inc.). The protein concentration was determined using Advanced protein assay reagent (Cytoskeleton).

### SDS-PAGE and analysis of IdeS activity

Proteins analyzed by SDS-PAGE were separated under reducing conditions on Nu-PAGE 4–12% Bis-Tris gels (Invitrogen) and stained with Coomassie Blue. Purified human IgG was purchased from European Institute of Science, purified rabbit IgG was purchased from Dakopatts, and blood samples were collected from healthy human donors. All activity assays with GST-IdeS (cleavage of IgG) were performed for 3 h at 37°C, and serum samples were diluted 50x in PBS prior to analysis by SDS-PAGE.

### Injections of IdeS into rabbits

Six Swedish loop rabbits (weighing approximately 5.0 kg) were injected i.v. with 5 mg GST-IdeS at day 0. Serum samples were collected from the ear vein at different time points and kept at −20°C until being analyzed for IgG and IdeS content. The procedure was repeated a second time at day 6 or at day 40 in all rabbits. To investigate possible toxic side effects of IdeS, all six rabbits were further injected up to six times with 5 mg GST-IdeS and visually monitored for signs of disease. After six intravenous injections, heart, lung, liver, spleen, and kidney tissue from two of the rabbits was subjected to histo-pathological analysis.

### IgG quantification in rabbit serum

To measure IgG clearance *in vivo*, rabbit serum samples were analyzed by SDS-PAGE and ELISA. In brief, Microtiter plates (Maxisorb, NUNC) were coated over night with rabbit serum samples diluted 5×10^4 ^times in coating buffer (0.05 M NaHCO_3_, pH 9.6). Plates were washed in PBST (0.05% Tween in PBS) and blocked with 2% bovine serum albumin (Sigma). IgG was detected using peroxidase-conjugated protein G (1∶3000, Bio-Rad) and the total amount of IgG in each sample was calculated by comparing the optical densites at 415 nm with a sequential dilution of rabbit IgG of known concentration. All analyses were performed in triplicates and the mean value ±SD for each sample was calculated.

### Determination of IdeS in rabbit serum

The circulation time of GST-IdeS in rabbit blood was determined by incubating 200 µl of rabbit serum samples with 40 µl Glutathione Sepharose 4B (Amersham Biosciences) for 60 minutes at 4°C. Following centrifugation, the pellets containing GST-IdeS and Glutathione Sepharose beads were boiled in 100 µl sample buffer and separated by SDS-PAGE. Samples were transferred to Immobilon-P transfer membranes (Millipore Inc.), and the extracted GST-IdeS was detected by rabbit serum containing specific anti-IdeS antibodies (1∶1000) as primary antibody followed by peroxidase-coupled Protein G (1∶3000, Bio-Rad). Incubations, performed for 1 h at 22°C, were followed by a washing step in PBST. GST-IdeS was visualized by chemiluminescence in a Chemidoc XRS imaging system, and the total amount of GST-IdeS present in the samples was calculated using Quantity One 1-D Analysis software, version 4.6 (Bio-Rad). The sensitivity of the assay is below 0.1 µg/ml serum. All analyses were performed in triplicates and the mean value±SD for each sample was calculated.

### Histo-pathological analysis

Rabbit organs were fixed in 4% paraformaldehyde (in PBS, pH 7.4) and processed for routine histo-pathological evaluation.

### IgG-induced thrombocytopenia in mice

Rabbit antiserum raised against mouse platelets was purchased from Intercell Technologies. IgG antibodies isolated from this serum were affinity purified on a Protein G column (Amersham Biosciences). Briefly, the column was prepared by washing with PBS and the rabbit antiserum was loaded onto the column. IgG bound to the column was eluted using acid glycine (0.1 M glycine, pH 2.0) and collected into neutralization buffer (1 M unbuffered Tris). IgG-containing fractions were pooled and the protein concentration was determined with SDS-PAGE and Advanced protein assay reagent (Cytoskeleton).

Female BALB/c mice (weighing approximately 20 g) were housed under standard conditions of light and temperature and were fed standard laboratory chow and water ad libitum. Rabbit anti-mouse platelet IgG was administered to the animals by i.p. injection. In pilot experiments 1.0 mg of purified IgG induced a lethal thrombocytopenia within 24 hours. Thirty-six mice were each injected i.p. with this dose of IgG (in 0.25 ml PBS). Thirty minutes or three hours later, the mice were treated with either an i.p. or an i.v injection of 0.5 mg IdeS diluted in 0.25 ml PBS, or with PBS alone. The animals were monitored for signs and symptoms of disease, and the survival time was recorded.

### Blood sampling from thrombocytopenic mice and platelet analyses

Immediately prior to the injection of rabbit anti-mouse IgG and at regular intervals during the course of the disease, blood samples were taken from the mice. The tail vein was pre-warmed and 5 µl of whole blood was collected into a tube containing 45 µl of citrate anticoagulant (0.11 M sodium citrate /citric acid in PBS, pH 6.5). The platelet population in these whole blood samples was identified using flow cytometry. Samples were labelled with hamster anti-mouse CD61 PE (BD Biosciences) for 10 minutes at room temperature. The red cell population was lysed using UtiLyse™ (Dako Cytomation) and the samples were analyzed on a FacsCalibur flow cytometer (BD Biosciences) in the logarithmic mode. The platelet population in the blood samples after lysis of the red blood cells, was also counted manually using phase contrast microscopy in a Neubauer chamber.

### Animal experiments

All animal studies conducted in this study have been approved by the ethical committee at Lund University.
